# The effects of oral nutritional supplements in patients with maintenance dialysis therapy: A systematic review and meta-analysis of randomized clinical trials

**DOI:** 10.1371/journal.pone.0203706

**Published:** 2018-09-13

**Authors:** Peng Ju Liu, Fang Ma, Qi Yan Wang, Shu Li He

**Affiliations:** Department of Clinical Nutrition, Peking Union Medical College Hospital, China Academic Medical Science and Peking Union Medical College, Beijing, PR China; Universidade Estadual Paulista Julio de Mesquita Filho, BRAZIL

## Abstract

**Background/objective:**

This systematic review aims to determine the potential effects of oral nutritional supplements (ONS) in patients receiving maintenance dialysis therapy (MDT).

**Methods:**

Electronic databases were searched without language limits through to July 2018. Randomized controlled trials (RCTs) that involved comparisons of ONS versus placebo or routine care are included in this meta-analysis. RevMan 5.3 statistical software was used for meta-analysis.

**Results:**

15 articles with 589 subjects were included in our study. There are insufficient comparable data of randomized trials to allow meta-analysis of mortality. Albumin levels may be improved by the macronutrient blends or protein/amino acid supplements in MDT patients. Compared with the control group, serum albumin levels and BMI in the ONS group were increased by 1.58 g/L (95% CI, 0.52–2.63, P = 0.003; I^2^ = 85%) and 0.40 kg/m^2^ (95% CI, 0.10–0.71, P = 0.01; I^2^ = 49%), respectively. In the subgroup analysis of patients receiving hemodialysis, albumin levels in ONS group were increased by 2.17 g/L (95% CI, 0.89–3.45, P<0.001; I^2^ = 90%). ONS may not influence serum phosphorus and potassium levels.

**Conclusions:**

Very low-quality evidence suggests that Short-term oral energy or protein/amino acid supplements may improve nutritional status by increasing serum albumin levels and BMI in MDT patients, without influence on serum potassium levels. High-quality and large RCTs, particularly regarding the effects of ONS on mortality and quality of life, are needed to further validate our findings.

## Introduction

Chronic kidney disease (CKD) is a prevalent chronic condition and the incidence of end-stage renal disease (ESRD) is expected to increase over the next few decades [1]. In patients with CKD, especially in those with ESRD and undergoing maintenance dialysis therapy (MDT), a state of metabolic and nutritional derangements, more aptly called protein-energy wasting (PEW), caused by a combination of insufficient intake, uremic toxins, inflammation, and superimposed catabolism [2,3], plays a major role among the many risk factors that affect outcomes of CKD [4]. PEW, showing a high prevalence (up to 50–75%) in patients with CKD stages 4–5, is closely associated with both increased morbidity/mortality risk and worsens quality of life [4]. Therefore, this term was introduced by the International Society of Renal Nutrition and Metabolism in 2008 to describe the status of decreased body stores of protein and energy fuels.

Several nutrition-related tests have been proposed to assess nutritional status in patients receiving MDT, such as subjective global assessment [5–7], the malnutrition inflammation score [8–10], body mass index (BMI) [11], serum albumin [12–14], and so on. Among these indicators, a low serum albumin level, which may indicate poor nutritional status and heightened inflammation, is a strong predictor of increased mortality risk [12,15–17], and appears to be better and simpler than others in reflecting nutritional status for patients receiving MDT[18,19].

In CKD, management of diet is important in prevention of disease progression and symptom management [20], and the evidence-based guidelines by British Dietetic Association have recommended that adults with maintenance hemodialysis or peritoneal dialysis require a minimum protein intake of 1.1 g/kg ideal body weight and 1.0–1.2 g/kg ideal body weight per day, respectively, in conjunction with an adequate energy intake [21]. However, patients undergoing MDT often suffer from anorexia and dysgeusia, which lead to inadequate protein and energy intake, resulting in poor nutritional status and adverse outcomes. Several studies have reported that dietary energy and protein intake were often lower than the recommendations for patients receiving MDT, especially on dialysis treatment days compared with non-dialysis treatment days [22,23]. Moreover, the consensus from International Society of Renal Nutrition and Metabolism stated that in patients where oral dietary intake from regular meals could not maintain adequate nutritional status, nutritional supplementation is shown to be effective in replenishing protein and energy stores [4]. Subsequently, patients may benefit from treatment of malnourishment [24] by supplementations of protein and energy [3,25], and improving the nutritional status of patients with CKD by nutritional support is expected to decrease morbidity and mortality. Oral nutritional supplement (ONS) is a simple and effective way to supplement energy and protein to malnourished patients on the basis of regular diet. Therefore, if the energy and/or protein of regular diet in dialysis patients are not enough, they should be supplemented with ONS when appropriate.

Although some reviews reporting nutritional supports for patients with CKD were published previously [26–30], they were either descriptive [26–29], or included a very small size of trials [30]. After those reviews, a number of randomized clinical trials (RCTs) have been published [31–43]. Therefore, our systematic review and meta-analysis of RCTs of patients with CKD undergoing MDT is undertaken with the following aim: to quantitatively examine the impact of oral nutritional supplementation of energy or protein/amino acid versus routine care on nutritional status, electrolyte level, and clinical outcomes.

## Materials and methods

### Search strategy

Four primary databases were searched without language limits up to July 2018: Pubmed (in the searching process, “Humans” and “Adult: 18+ years” were used as filters), Embase, clinicalTrials.gov, and Cochrane Library. Search terms included dialysis, hemodialysis, haemodialysis, chronic renal failure, nutrition*, oral supplement*, ONS, nutrient*, macronutrients, calorie supplement*, energy supplement*, protein supplement*, amino acid supplement*. In addition, the reference lists of the published papers on clinical trials, review articles and meta-analysis were handed-searched for other relevant studies. The complete search strategy is available in the Table 1.

**Table 1:** Summary of inclusion and exclusion criteria applied during evaluation of studies for systematic review.

Selection criteria	Inclusion criteria	Exclusion criteria
Population	Adults studies; Nutrition status(either well nourished or malnourished); Patients undergoing dialysis (of any type)	Animal data
Intervention	All studies using oral nutritional supplements with any macronutrient (carbohydrate, fat, or protein/amino acid); Setting in hospital or community (outpatient or home)	Feeds only given non-caloric nutrients or concomitantly given keto acid or keto analogues
Comparison	Placebo, routine care, or no supplementation	Without control group
Outcome measures	Mortality; Serum albumin level; Body mass index; Electrolytes (serum potassium and phosphate)	Studies without any predetermined outcome measure
Study type	Randomized controlled trials	Non-randomized studies

### Study selection criteria

RCTs are included if they met the following criteria: (1) the study compared energy (macronutrient blends) or protein/amino acid supplementations (any dose or type) with or without micronutrients versus placebo/no treatment (standard care); (2) at least one of the following outcomes were reported: serum albumin, BMI, phosphorus and potassium, mortality, quality of life; (3) all patients > 18 years; and (4) interventions were given through oral nutritional supplements, which could be a commercial or a non-commercial (food mixture or liquid homogenate obtained by crushing food) nutrition supplement.

### Data extraction and quality assessment

Two reviewers (L. P. J and W. Q. Y) independently analyzed the titles and abstracts of every paper retrieved from the literature search to identify potentially eligible studies. The full text of the remaining papers was obtained for further examination. Differences in interpretations were resolved through discussion.

Eligible studies were reviewed independently by the same two reviewers who used a standardized data extraction forms developed for this purpose. Data were extracted from all eligible trials with the following reported outcomes: serum albumin, BMI, phosphorus and potassium, mortality, quality of life. If the study was a crossover study, outcomes at the end of the first phase (before the crossover) were then used.

Another two reviewers (M. F. and H. S. L) independently assessed the methodological quality of studies based on the Jadad quality scale [44], which graded the quality of a study from 0 (lowest) to 5 (highest) by examining randomization, blind, and follow-up (withdrawals and dropouts).

### Data synthesis and statistical analysis

For continuous variables, if the mean and SDs of the changes from baselines were given in the studies, they were then directly used. Otherwise, the mean changes were calculated by subtracting the baseline values from the final values, and the SD of the difference was calculated as following formula (which was based on the study by Pei J et al. [45]):

$$SD={\left[{\left(SD1\right)}^{2}+{\left(SD2\right)}^{2}-2R*SD1*SD2\right]}^{0.5}\left(R=0.5\right)$$

If there were two intervention groups of the same nature in one study, they were then merged as one whole intervention group through the following method:

N(merged)=N1+N2

Mean(merged)=(Mean1*N1+Mean2*N2)/(N1+N2)

$$SD\;\left(merged\right)={\left\{\left(N1-1\right)*{\left(SD1\right)}^{2}+\left(N2-1\right)*{\left(SD2\right)}^{2}+{\left(Mean1-Mean2\right)}^{2}\;*\left[N1*N2/\left(N1+N2\right)\right]\right\}}^{0.5}/{\left\{N1+N2-1\right\}}^{0.5}$$

Review Manager 5.3 statistical software (Cochrane Collaboration) was used for meta-analysis. For dichotomous outcomes, the results were analyzed using relative risk (RR) with a 95% confidence interval (95% CI), while continuous data were analyzed using mean differences (MDs) or standardized mean differences (SMDs), when appropriate. Publication bias was assessed by visual inspection of a funnel plot and the Egger’s test was also performed to assess its possibility. Heterogeneity among studies was evaluated using I^2^ statistic. I^2^ > 50% is considered significant. Given the potentially clinical heterogeneity between the included trials due to their differences in the timing of intervention (intradialytic supplementation or general supplementation), type of dialysis (hemodialysis or peritoneal dialysis), intervention duration, albumin levels and nutritional status of the included patients, and type of ONS (multinutrients or protein/amino acid), the random effects model was chosen.

Given the potential heterogeneity described above, the subgroup analyses about albumin compared patients stratified by the timing of intervention, the type of dialysis, the duration of intervention, the levels of albumin, and the type of ONS. Sensitivity analyses were performed by omitting one study at a time and analyzing the remaining studies to assess whether the results were excessively influenced by any single study. Moreover, we further divided the included trials into low-quality (non-blinded) and high-quality (double-blinded) studies, and then we observed the change of heterogeneity.

## Results and discussion

### Literature search and quality assessment

**Fig 1:**
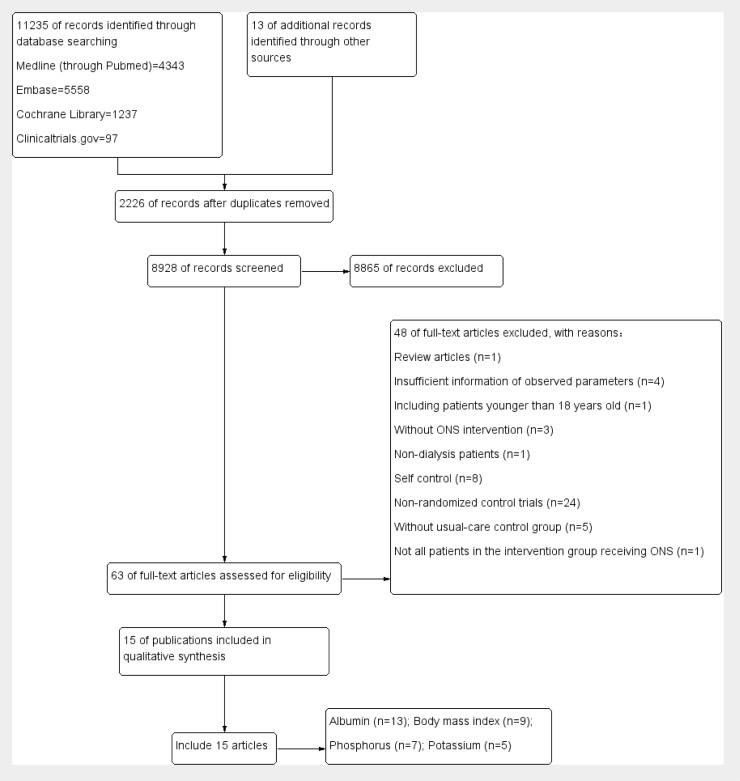
Flow diagram of study selection process.

A total of 11235 articles were identified by the search strategy (Fig 1). After removal of duplicate, 8928 records were left. Of these, 8865 were excluded based on title and abstract, leaving 63 articles for further full text evaluation. Of these articles, one randomized trial was excluded for its inappropriate age range of the participants [42]. Finally, we identified 15 articles with 589 subjects in our study [31–41,43,46–48]. Among these publications, although there was a study in which the age range of the subjects were from 17 to 65 years [39], we eventually included this study through discussion, as the clinical characteristics of people who aged 17 years old are very close to those of the adults and that study showed a high quality according to the Jadad score. The characteristics of the included studies are shown in Table 2, and quality assessment of the included studies is summarized in Table 3. As shown in Table 3, only three trials [31,41,47] scored 2 in the assessment of blinding according to Jadad scale, and the remaining trials [32–40, 43, 46,48] scored 0 (lowest), which is the most important factor contributing to a high overall risk of bias. Similarly, when assessed randomization, less than half of the included trials [35,39–41,43,47] reached a full score (2 points), while the remaining [31–34,36–38,46,48] only scored 1 point, resulting in an unclear risk of selection bias, which might be at least in part associated with an overall risk of bias. In addition, all reported their follow-ups (withdrawals and dropouts) except one trial [35], showing a low risk of reporting bias. To be noted, there was one study in which the magnitude of the unit of albumin appeared to be with some errors (the unit of albumin was presented as “mg/dl”) [31], leading to an unclear risk of other bias.

**Table 2:** Characteristics of the trials included in the meta-analysis.

Reference (published year)	Patients (n) in each group	Population Description	Intervention modality of case group	Intervention modality of control group	Study design and duration	Main outcomes measures
Tomayko EJ et al. (2015) [31]	Case (11 and 12); control (15)	MHD patients, treatment for ≥ 3 months, ≥ 3 days/week, nutritional status was not stated	27 g whey or 27 soy protein within beverage, consumed within 15 mins of dialysis; intradialytic supplementation	2g non-caloric powder within beverage, consumed within 15 mins of dialysis	Randomized, controlled, blinded; 6 mo	Albumin, quality of life, phosphorus, and potassium
Calegari A et al. (2011) [32]	Case (9); control (6)	Malnourished HD patients (SGA >15 points)	Non-industrialized nutrition supplement (Thick mixed food), 355 kcal, 53% of carbohydrate, 10g of protein, and 15g of lipid; intradialytic supplementation	Routine nutritional guidance	Randomized,controlled, non-blinded,crossover; 3 mo (first phase)	Body mass index, albumin, quality of life, phosphorus, and potassium
Bolasco P et al. (2011) [33]	Case (15); control (14)	HD patients, thrice-weekly albumin < 3.5 g/dL and BMI > 20 kg/m^2^, nutritional status was not stated	12 g amino acid powder dissolved in water; general supplementation	No intervention	Randomized,controlled, non-blinded;3 mo	Body mass index, albumin
Tabibi H et al. (2010) [34]	Case (18); control (18)	Continuous ambulatory PD; nutritional status was not stated	28g packets of raw textured soy flour (containing 14g of soy protein); general supplementation	Usual diet without consumption of soy-containing products	Randomized,controlled, non-blinded;8 weeks	Albumin
Imani H et al. (2009) [35]	Case (18); control (18)	Continuous ambulatory PD; nutritional status was not stated	28g packets of raw textured soy flour (containing 14g of soy protein); general supplementation	Usual diet without consumption of soy-containing products	Randomized,controlled, non-blinded;8 weeks	Phosphorus
Fouque D et al. (2008) [36]	Case (37); control (29)	MHD patients, albumin < 40 g/L and BMI <30 kg/m^2^; mildly nourished	250 ml Renilon 7.5 daily, 500 kcal, containing 18.75 g protein and 15 mg phosphotus; general supplementation	Standard care	Multicentre, randomized,open-label, controlled; 3 mo	Body mass index, albumin, and quality of life
González-Espinoza L et al. (2005) [37]	Case (13); control (15)	Continuous ambulatory PD for at least 1 month; malnourished	22g of high biological-value protein (egg albumin) daily; general supplementation	Conventional nutritional counseling	randomized,open-label, controlled; 6 mo	Albumin, phosphorus, and potassium
Sharma M et al. (2002) [47]	Case (16 and 10); control (14)	Malnourished; regular thrice weekly MHD patients(for at least 1 month) and BMI<20kg/m^2^ and albumin < 4.0g/dL	Standard home-prepared ONS: (500 kcal and 15g protein) vs CKD-specific ONS (Reno care II, Criticare, Mumbai, India (500 kcal and 15g protein); general supplementation	Dietary counseling no specific post-HD supplement	Randomized,controlled, non-blinded;1 mo	Body mass index, albumin, quality of life, potassium and phosphorus
Sohrabi Z et al. (2016) [39]	Case (23): control (23)	Regular HD patients with malnutrition	15g whey protein without vitamin E (three times per week); intradialytic supplementation	No intervention	Randomized,controlled, non-blinded;8 weeks	Body mass index, albumin, and phosphorus
Hung SC et al. (2009) [40]	Case (20); control (21)	Nondiabetic HD patients; malnourished	Daily use of one can of a commercially ONS (475 kcal, contained 16.6g protein, 22.7g fat, and 52.8g carbohydrate); general supplementation	Without supplementation	Prospective, randomized, controlled, non-blinded; 12 weeks	Body mass index, albumin
Eustace JA et al. (2000) [46]	HD: Case (14); control (15) PD: Case (9); Control (9)	HD and PD patients, albumin <3.8g/dL); nutritional status was not stated	Daily 10.8g EAA with meals; general supplementation	Placebo in appearance to the EAA tablets	Randomized,double-blind, controlled; 3 mo	Body mass index and albumin
Morretti HD et al. (2009) [38]	Case (31); Control (18)	HD and PD patients; nutritional status was not stated	15g liquid hydrolyzed collagen protein (3 times per week for HD patients and 7 times per week for PD patients) general supplementation	No supplement	Randomized,controlled, non-blinded, crossover; 6 mo	Albumin
Allman MA et al. (1990) [45]	Case (9)/Control (12)	Regular HD patients for > 3 months; nutritional status was not stated	100-150g glucose-polymer(400-600kcal) plus water-soluble vitamin; general supplementation	No energy supplement	Randomized controlled, non-blinded;6 mo	Body mass index and albumin
Rattanasompattikul M et al. (2013) [41]	Case (22); Control (21)	MHD patients with Alb < 40 g/L; nutritional status was not stated	19 g protein combined with fish oil, borage oil, beta-carotene, vitamin C and E, zinc, and selenium; intradialytic supplementation	Placebo	Randomized,double-blind,controlled; 16 weeks	Albumin, phosphorus, and potassium
Sahathevan S, et al. (2018)[43]	Case(37);control(37)	Malnourished peritoneal dialysis patients, with Alb<40 g/L and BMI<24.0 kg/m^2^	27.4g whey protein powder ingested post-meal plus dietary counseling	dietary counseling only	Randomized,controlled, open-label 6 mo	Albumin, BMI, phosphorus, and quality of life

MHD, maintenance hemodialysis; HD, hemodialysis; PD, peritoneal dialysis.

**Table 3:** Quality assessment of the included randomized controlled trials.

Reference	Total score	Randomization	Blind	Follow-up	Data source
Tomayko EJ et al. (2015) [31]	4	1	2	1	Paper
Calegari A et al. (2011) [32]	2	1	0	1	Paper
Bolasco P et al. (2011) [33]	2	1	0	1	Paper
Tabibi H et al. (2010) [34]	2	1	0	1	Paper
Imani H et al. (2009) [35]	2	2	0	0	Paper
Fouque D et al. (2008) [36]	2	1	0	1	Paper
González-Espinoza L et al. (2005) [37]	2	1	0	1	Paper
Sharma M et al. (2002) [47]	2	1	0	1	Paper
Sohrabi Z et al. (2016) [39]	3	2	0	1	Paper
Hung SC et al. (2009) [40]	3	2	0	1	Paper
Eustace JA et al. (2000) [46]	5	2	2	1	Paper
Morretti HD et al. (2009) [38]	2	1	0	1	Paper
Rattanasompattikul M et al. (2013) [41]	5	2	2	1	Paper
Sahathevan S, et al. (2018)[43]	3	2	0	1	Paper
Allman MA et al. (1990) [45]	2	1	0	1	Paper

Among the trials included, there were differences in the timing of intervention (intradialytic supplementation or general supplementation), type of dialysis (hemodialysis or peritoneal dialysis), intervention duration, albumin levels and nutritional status of the included subjects, and type of ONS. In four trials [31,32,39,41], nutritional supplementations were administrated during dialysis (intradialytic supplementation), while the others [33–38,40,43,46–48] were not (general supplementation). For type of dialysis, hemodialysis patients and peritoneal dialysis patients were recruited in nine [31–33,39–41,46–48] and three trials [34,37,43,47], respectively. As for albumin levels, the patients of four trials [33,36,43,47,48] were with albumin level <40 g/L, while the others [31,32,34,35,37–41,46] were without specific descriptions. For the type of ONS, protein/amino acid were administrated in 9 trial (10 publications) [31,33–35,37–39,41,43,47] and multinutrients were used in four trials [32,36,40,48]. In addition, seven studies [32–34,39,40,47,48] had an intervention duration of ≤ 3 months and six studies [31,37,38,41,43,46] were conducted for a duration of > 3 months.

### Effects of ONS on serum albumin

**Fig 2:**
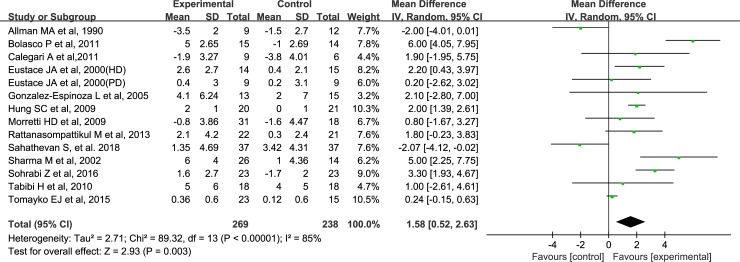
Forest plots depicting the effect of ONS on serum albumin level.

The effects of ONS on the serum albumin in all studies are shown in Fig 2. A total of 269 MDT patients were allocated to the supplementary group, whereas 238 MDT patients constituted the control group. Overall, the pooled analysis of the effects of ONS on albumin levels shows an increase of 1.58 g/L compared with the control group (95% CI, 0.52–2.63, P = 0.003), and a significant degree of heterogeneity is observed for this outcome (I^2^ = 85%, P<0.0001) (Fig 2). The overall estimated effect of ONS on serum albumin, together with the corresponding quality of evidence, is presented in **S1 Table**.

#### Subgroup analysis of the effects of ONS on albumin

Given the expected clinical heterogeneity, the results of the subgroup analyses are performed and shown in Table 4. In the subgroup analysis, the similar trends of ONS on serum albumin are found for patients with albumin level < 40 g/L [2.19 g/L (-0.23, 4.60), 95% CI, I^2^ = 87%, P = 0.08] and patients with all possible levels [1.12 g/L (-0.07, 2.30), 95% CI, I^2^ = 84%, P = 0.07].

**Table 4:** Results of subgroup analyses about the effects of ONS on albumin.

Subgroup		Albumin (g/L)			
		Effect size	95% CI	I^2^	P value
Type of dialysis	Hemodialysis (n = 9)	2.17	0.89, 3.45	90%	<0.001
	Peritoneal dialysis (n = 4)	-0.63	-2.07, 0.81	29%	0.39
Intervention duration	≤3 months (n = 7)	2.45	1.96, 2.94	69%	<0.0001
	>3 months (n = 6)	0.11	-0.26, 0.48	60%	0.56
Timing of supplementation	Intradialytic (n = 4)	1.73	-0.18, 3.64	85%	0.08
	General (n = 9)	1.51	-0.03, 3.05	83%	0.05
Albumin levels	< 40 g/L (n = 5)	2.19	-0.23, 4.60	87%	0.08
	All possible levels (n = 8)	1.12	-0.07, 2.30	84%	0.07
Type of ONS	Protein/amino acid (n = 9)	1.58	0.17, 2.99	85%	0.03
	Multinutrients (n = 3)	2.78	0.86, 4.71	54%	0.005

ONS, oral nutritional supplements; CI, confidence interval.

According to the intervention duration, studies were divided into long-term group (> 3 months) and short-term group (≤ 3 months), and the subgroup analysis showed that prolonged intervention did not appear to improve albumin levels; However, short-term intervention appeared to improve albumin levels [2.45 g/L (1.96, 2.94), 95% CI, I^2^ = 69%, P<0.0001].

Based on the included studies, the patients from 9 trials received hemodialysis, whereas patients of 4 trials underwent peritoneal dialysis. The results of meta-analysis show that ONS significantly improves the albumin levels in patients undergoing hemodialysis [2.17 g/L (0.89, 3.45), 95% CI, I^2^ = 90%, P<0.001], but not in patients receiving peritoneal dialysis.

Moreover, the total included patients were divided into macronutrient blends group and protein/amino acid group according to the type of intervention. The results show that supplementations with both of the macronutrient blends and protein/amino acid could improve albumin levels in patients undergoing MDT.

### Effects of ONS on BMI

**Fig 3:**
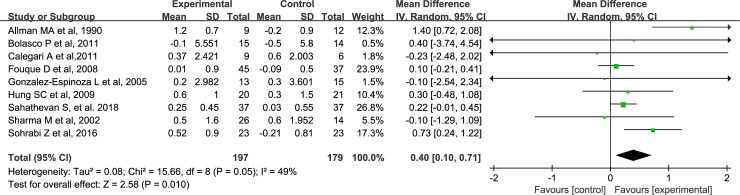
Forest plots depicting the effect of ONS on body mass index.

Compared with the control group, BMI in the ONS group are increased by 0.40 kg/m^2^ (95% CI, 0.10–0.71, P = 0.01), and the heterogeneity of the result is not significant (I^2^ = 49%, P = 0.05) (Fig 3). The overall estimated effect of ONS on BMI, together with the corresponding quality of evidence, is presented in **S1 Table**.

### Effects of ONS on phosphorus and potassium levels

**Fig 4:**
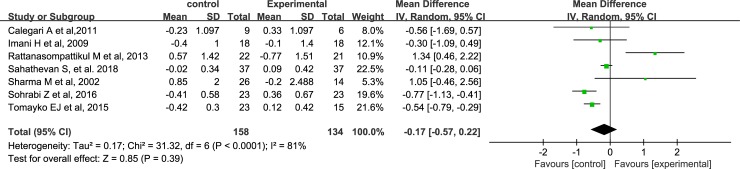
Forest plots depicting the effect of ONS on serum phosphorus.

**Fig 5:**
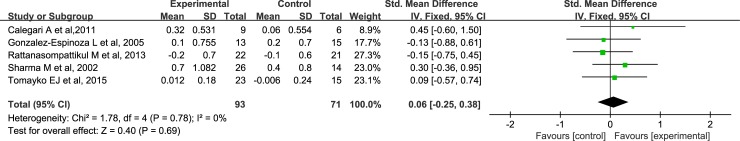
Forest plots depicting the effect of ONS on serum potassium.

A total of 7 studies and 5 studies were included in the comparison of the effects of ONS on phosphorus levels and potassium levels, respectively. The pooled analysis shows that ONS do not influence serum phosphorus [-0.17 mg/dl, 95% CI, (-0.57, 0.22), P = 0.39] (Fig 4) and potassium levels [0.06, 95% CI, (-0.25, 0.38), P = 0.69] (Fig 5) in dialysis patients.

### Publication bias

**Fig 6:**
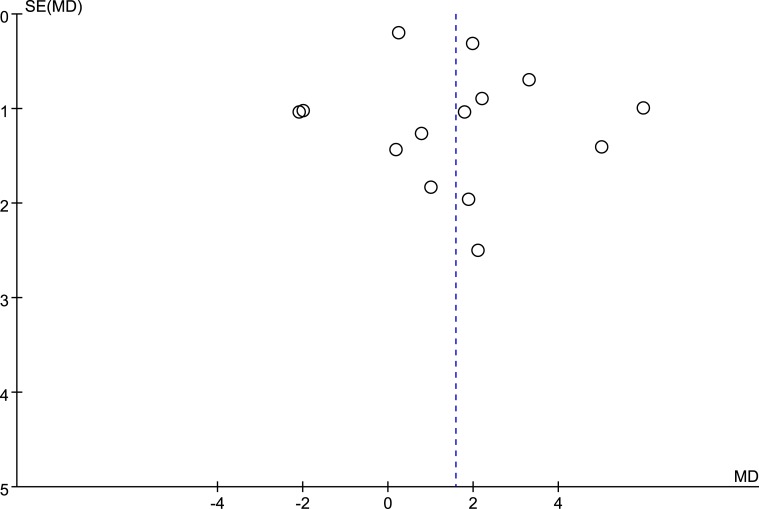
Funnel plots of studies included in meta-analysis on the effects of ONS on serum albumin level.

The potential publication bias was detected by funnel plots (Fig 6) and Egger’s test. The results suggest no publication bias for the effects of ONS on the serum albumin. Besides albumin, we do not draw the funnel plots for the meta-analysis of other parameters due to their small size of the included studies (n < 10).

### Effects of ONS on mortality or quality of life

Based on the current included studies, there are insufficient comparable data of randomized trials to allow meta-analysis of mortality. However, several trials provided the reports of quality of life that could be considered as a clinical outcome. In the study by Fouque et al. [36], there was an improvement for the general health and bodily pain components, parts of the assessment for quality of life, in hemodialysis patients who effectively took the renal-specific oral supplement. In another trial [48], the authors reported that ONS could result in an improvement in functional scores (Karnofsky Index scores) in both intervention groups, that is, MDT patients receiving the supplement often reported a subjective sense of well-being with amelioration of postdialysis fatigue. However, the data on quality of life could not be pooled due to their obvious differences.

### Sensitivity analysis

We found an obvious heterogeneity in our analysis of the effect of ONS on albumin. We then used method of “exclusion of one trial each time”. However, the change of heterogeneity was not obvious for each exclusion (data were not shown). After that, the heterogeneity was decreased to 53% after further exclusion of non-blinded trials [32–34, 36–40,43,46]. Notably, in the double-blinded trials, there was one study in which the magnitude of the unit of albumin appeared to be with some errors (the unit of albumin was presented as “mg/dl”) [31], thus, we then further excluded this trial to analyze its influence and found that the heterogeneity was reduced to 0% with a significant increase of albumin level [1.58 g/L (0.52–2.63), 95% CI, P = 0.003].

This analysis is a systematic review of randomized controlled trials investigating the effects of ONS in the patients undergoing maintenance dialysis therapy (MDT). Our results support the use of ONS for the improvement of nutritional status in MDT patients without significant influence on serum phosphorus and potassium levels, especially for patients receiving hemodialysis. Our conclusion is consistent with a previous review [30] which included a very small size of trials and was published over 10 years ago about this area. However, an overall heterogeneity and a very low-quality evidence is identified in our study, therefore the results of our meta-analysis should be interpreted in a cautious manner, and the suggestion given to patients with MDT should be based on their specific conditions.

PEW is highly prevalent in patients undergoing MDT, and one frequent and important cause is an inadequate dietary protein and energy intake [49–51]. Furthermore, additional nutrient loss during dialysis such as amino acid, some peptides, blood, vitamins, trace elements, and glucose may further predispose these patients to an increased risk of PEW [52,53], which is closely associated with the increased mortality risk in dialysis patients. A low serum albumin concentration is by far the strongest predictor of poor outcomes and mortality in dialysis patients when compared with any other risk factor. The association between serum albumin levels and mortality is highly incremental and linear, and the mortality predictability of a serum albumin concentration of <40 g/l has virtually no cut-off level below which the association with death would cease or reverse according to Kalantar-Zadeh K et al. and Lacson E et al. [12,54].

Mortality rates are high among hemodialysis patients, with almost 50% dying within 3 years of initiating kidney replacement therapy [55]. While numerous epidemiological data suggest that an improvement in biomarkers of nutritional status is associated with improved survival, there are no large randomized clinical trials that have tested the effectiveness of nutritional interventions on mortality and morbidity [4]. Currently, we can only obtain the relevant data from retrospective and cohort study [20,56] and the quality of the body of evidence is poor [20].

Our meta-analysis shows that oral supplementation with energy or protein/amino acid is associated with an improvement on nutritional status by resulting in an increase in serum albumin level. According to Kalantar-Zadeh K et al. and Lacson E et al. [12,54], the sensitivity of measuring serum levels of albumin to predict outcomes in patients with CKD is high, with a granularity of as little as 2 g/L or less. In other words, a patient on dialysis with a baseline serum albumin concentration of 2 g/L above or below that of another patient with similar demographic features and comorbidities has a substantially decreased or increased risk of death, respectively. Although there are insufficient comparable data to allow meta-analysis of mortality based on the included studies, the results of our meta-analysis for albumin might, to some extent, suggest that ONS is likely to improve the mortality risk in dialysis patients by improving albumin levels. In addition, several studies have reported that ONS significantly improves functional status and quality of life during the course of the study [31,32,36,48]. However, the data on quality of life could not be pooled due to their obvious differences.

In the present meta-analysis about the overall effects of ONS on albumin, an obvious heterogeneity is identified. We subsequently compare subgroups to analyze the heterogeneity. In the subsequent subgroup analyses, stratified by the albumin level, intervention duration, timing of supplementation, type of intervention and type of dialysis, it is found that both of the macronutrient blends and protein/amino acid supplementation could improve albumin levels in patients receiving MDT. As for the timing of supplementation, we find that general supplementation improved the albumin levels but not intradailytic supplementation. In addition to a small sample size, another potential reason for the result might be that, in general-supplementation group, most of the supplementary methods are on a daily basis, whereas in intradialytic supplementation group the methods of supplementations are all based on three times a week, the patients in general-supplementation group subsequently obtain more nutrients than those in intradialytic supplementation group. Therefore, more relevant studies are needed to investigate the differences between the timing of supplementations. Additionally, prolonged ONS (> 3 months) do not improve serum albumin levels; however, short-term supplements intervention (≤ 3 months) appears to improve albumin levels. The reasons for these results might be due to a relatively small number of long-term studies and the different baseline levels of albumin between short-term group and long-term group. Therefore, additional long-term well-designed RCTs are needed to be performed.

Besides the findings above, we found that the heterogeneity still exists in most of the subgroup analyses, and we then used method of “exclusion of one trial each time”, similarly, the change of heterogeneity was not obvious for the exclusion each time. However, after the exclusion of all non-blinded trials [32–34, 36–40,43,46], the heterogeneity was decreased to 53%. When the study by Tomayko et al. [31] (in which the magnitude of the unit of albumin appeared to be with some errors [the unit of albumin was presented as “mg/dl”]) was further excluded, despite the small number of remaining trials, we found that the heterogeneity was reduced to 0% in the remaining double-blinded trials, with a significant increase of albumin level [1.58 g/L (0.52–2.63), 95% CI, P = 0.006] by ONS, which indicates that the obviously overall heterogeneity for analyses about the effects of ONS on albumin is due to the very-low quality of the most of the trials included. Therefore, more high-quality trials are needed to further demonstrate our findings.

BMI is another marker reflecting nutritional status in patients with MDT. As for the association of BMI with the mortality, conflicting results have been reported among the peritoneal dialysis (PD) patients [57–59]. A most recent meta-analysis has reported that a high BMI (obesity) is associated with increased mortality in Asian PD patients and also indicated a “V-shaped” trend in the association between BMI and mortality in those patients [60]. In hemodialysis (HD) patients, a high BMI is paradoxically associated with a better outcome, and this paradoxical association between BMI and mortality can be modified by inflammation [61]. In our meta-analysis, we find that BMI in the ONS group were increased by 0.40g/m^2^ when compared with control group, suggesting that ONS may improve nutritional status in patients receiving MDT.

There are insufficient data to analyze the causal relationship between ONS and mortality, and it can not be elucidated that an improvement of BMI is associated with the better outcomes. Therefore, further studies are expected.

Additionally, our review find that oral supplements with energy or protein/amino acid have no significant effect on serum potassium level as well as phosphorus level, which is consistent with one previous review [30]. However, the phosphorous results have high heterogeneity, and the possible reason might be due to the clinical heterogeneity between the included studies, which might suggest that more similar studies need to be conducted to confirm the effects of ONS on phosphorus level.

Our review has several limitations. Firstly, the number of the included studies was relatively small due to the limited current data and most of them were single-center studies with the small sample size; moreover, the overall quality of the included trials is very low; Secondly, a significant heterogeneity for the meta-analysis of the effects of ONS on serum albumin levels was observed in our current study, thus we have to be cautious in interpreting the results of the analysis; Thirdly, based on current data of the included studies, we could not determine the effects of ONS or a short-term increase in albumin level on the mortality risk in dialysis patients; Finally, we could not evaluate the effects of ONS on inflammation, which is closely associated with the outcomes in patients receiving MDT.

## Conclusions

In conclusion, evidence of very-low quality suggests that short-term oral nutritional supplements with energy or protein/amino acid were found to be associated with increased albumin level, especially in those who receiving hemodialysis. More high-quality and large RCTs, particularly those involving the observation of mortality and/or quality of life, are needed to validate our findings in a long-term way.

## Supporting information

S1 File PRISMA 2009 checklist. (DOC)

S1 Table The GRADE summary of findings about the overall effects of ONS on albumin and BMI together with the quality of evidence. (DOCX)
