# Supplementary material for: The effects of oral nutritional supplements in patients with maintenance dialysis therapy: A systematic review and meta-analysis of randomized clinical trials
Source: PLoS One. 2018 Sep 13;13(9):e0203706. doi: 10.1371/journal.pone.0203706 (PMC6136747; doi:10.1371/journal.pone.0203706)
Supplement: S1 Table — (DOCX) [file pone.0203706.s002.docx]

| **ONS compared to control or standard care for dialysis** | | | | | | |
| --- | --- | --- | --- | --- | --- | --- |
| **Patient or population:** patients with dialysis **Settings:** Regular dialysis in hospitals or families **Intervention:** ONS；**Comparison:** control or standard care | | | | | | |
| **Outcomes** | **Illustrative comparative risks* (95% CI)** | | **Relative effect (95% CI)** | **No of Participants (studies)** | **Quality of the evidence (GRADE)** | **Comments** |
|  | Assumed risk | Corresponding risk |  |  |  |  |
|  | **Control or standard care** | **ONS** |  |  |  |  |
| **Effects of ONS on BMI** |  | The mean effects of ons on bmi in the intervention groups was **0.4 higher** (0.1 to 0.71 higher) |  | 376 (9 studies) | ⊕⊕⊝⊝  **low**^1^ |  |
| **Effects of ONS on albumin level** |  | The mean effects of ons on albumin level in the intervention groups was **1.58 higher** (0.52 to 2.63 higher) |  | 507 (14 studies) | ⊕⊝⊝⊝  **very low**^2,3^ |  |
| *The basis for the **assumed risk** (e.g. the median control group risk across studies) is provided in footnotes. The **corresponding risk** (and its 95% confidence interval) is based on the assumed risk in the comparison group and the **relative effect** of the intervention (and its 95% CI). **CI:** Confidence interval; | | | | | | |
| GRADE Working Group grades of evidence **High quality:** Further research is very unlikely to change our confidence in the estimate of effect.  **Moderate quality:** Further research is likely to have an important impact on our confidence in the estimate of effect and may change the estimate. **Low quality:** Further research is very likely to have an important impact on our confidence in the estimate of effect and is likely to change the estimate. **Very low quality:** We are very uncertain about the estimate. | | | | | | |
| ^1^ 6 studies out of 9 included in the review rated 2 out of 5 according to Jadad quality scale ^2^ 7 studies out of 14 included in the review rated 2 out of 5 according to Jadad quality scale ^3^ Heterogeneous results with inconsistency | | | | | | |
